# Poly[μ_3_-hydroxido-μ-(pyridine-2,4,6-tricarboxyl­ato)-dilead(II)]

**DOI:** 10.1107/S1600536810049275

**Published:** 2010-12-04

**Authors:** Ying-Hua Zhou, Jian Chen, Yong Cheng, Nian-Rong Zhang

**Affiliations:** a College of Chemistry and Materials Science, Anhui Normal University, Wuhu 241000, People’s Republic of China

## Abstract

The asymmetric unit of the title coordination polymer, [Pb_2_(C_8_H_2_NO_6_)(OH)]_*n*_, contains two crystallographically independent Pb^II^ ions, one pyridine-2,4,6-tricarboxyl­ate (ptc) trianion and one hydroxide anion. One of the Pb^II^ atoms is coordinated by one pyridine N and four carboxyl­ate O atoms from the ptc trianion and a hydroxide O atom in a distorted octa­hedral geometry. The other Pb^II^ atom is five-coordinated by three carboxyl­ate O atoms and two hydroxide O atoms in a distorted tetra­gonal–pyramidal geometry. Four neighbouring Pb^II^ atoms are bridged through two μ_3_-hydroxide ligands, forming the centrosymmetric Pb_4_(OH)_2_ core. The three-dimensional structure is further achieved through bridging carboxyl­ate groups. There are also O—H⋯O hydrogen bonds between the hydroxide ligand and the carboxyl­ate group.

## Related literature

For general background to pyridine-2,4,6-tricarb­oxy­lic acid complexes and their derivatives, see: Das *et al.* (2009 ▶); Ding *et al.* (2009 ▶); Ghosh *et al.* (2006 ▶); O’Keeffe *et al.* (2008 ▶); Shi *et al.* (2010 ▶); Xu *et al.* (2010 ▶); Yigit *et al.* (2005 ▶); Zhang *et al.* (2009 ▶); Zhao *et al.* (2009 ▶). For our previous work on metal complexes, see: Zhou *et al.* (2007 ▶); Wu *et al.* (2007 ▶). 
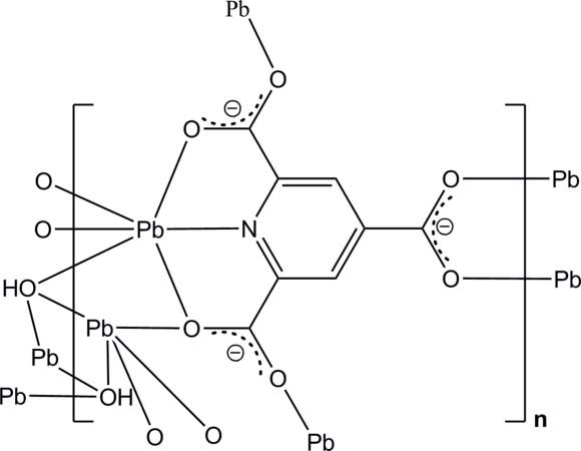


## Experimental

### 

#### Crystal data

[Pb_2_(C_8_H_2_NO_6_)(OH)]
*M* *_r_* = 639.49
Monoclinic, 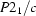

*a* = 7.5391 (9) Å
*b* = 14.1845 (17) Å
*c* = 10.3084 (12) Å
β = 100.468 (1)°
*V* = 1084.0 (2) Å^3^
*Z* = 4
Mo *K*α radiation
μ = 31.05 mm^−1^
*T* = 291 K
0.38 × 0.26 × 0.25 mm

#### Data collection

Bruker SMART APEX CCD area-detector diffractometer
Absorption correction: multi-scan (*SADABS*; Sheldrick, 1996 ▶) *T*_min_ = 0.030, *T*_max_ = 0.047
7870 measured reflections
2014 independent reflections
1903 reflections with *I* > 2σ(*I*)
*R*_int_ = 0.036

#### Refinement

*R*[*F* ^2^ > 2σ(*F* ^2^)] = 0.028
*wR*(*F* ^2^) = 0.069
*S* = 1.08
2014 reflections
157 parameters
H-atom parameters constrained
Δρ_max_ = 1.64 e Å^−3^
Δρ_min_ = −2.07 e Å^−3^

### 

Data collection: *SMART* (Bruker, 1997 ▶); cell refinement: *SAINT* (Bruker, 1997 ▶); data reduction: *SAINT*; program(s) used to solve structure: *SHELXS97* (Sheldrick, 2008 ▶); program(s) used to refine structure: *SHELXL97* (Sheldrick, 2008 ▶); molecular graphics: *SHELXTL* (Sheldrick, 2008 ▶); software used to prepare material for publication: *SHELXTL*.

## Supplementary Material

Crystal structure: contains datablocks I, global. DOI: 10.1107/S1600536810049275/is2628sup1.cif

Structure factors: contains datablocks I. DOI: 10.1107/S1600536810049275/is2628Isup2.hkl

Additional supplementary materials:  crystallographic information; 3D view; checkCIF report

## Figures and Tables

**Table 1:** Selected bond lengths (Å)

Pb1—O5^i^	2.422 (6)
Pb1—O1	2.489 (6)
Pb1—N1	2.554 (6)
Pb1—O7^ii^	2.627 (5)
Pb1—O3^iii^	2.697 (6)
Pb1—O6	2.716 (6)
Pb2—O1	2.600 (6)
Pb2—O2^iv^	2.836 (7)
Pb2—O4^v^	2.541 (6)
Pb2—O7	2.318 (5)
Pb2—O7^ii^	2.393 (5)

Symmetry codes: (i) 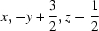; (ii) 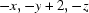; (iii) 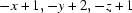; (iv) 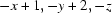; (v) 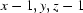.

**Table 2:** Hydrogen-bond geometry (Å, °)

*D*—H⋯*A*	*D*—H	H⋯*A*	*D*⋯*A*	*D*—H⋯*A*
O7—H7⋯O6^vi^	0.83	2.58	2.989 (8)	112
O7—H7⋯O4^iii^	0.83	2.49	2.884 (8)	110

Symmetry codes: (iii) 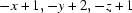; (vi) 
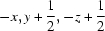
.

**Fig. 1.**
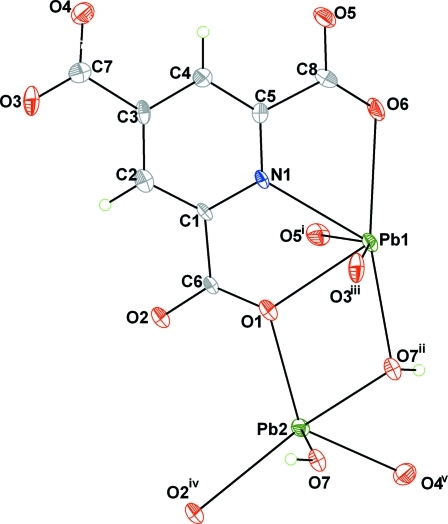
A view of the local coordination of the PbII atoms in the title compound, showing the atom-numbering scheme. Displacement ellipsoids are drawn at the 50% probability level. H atoms are shown as small spheres of arbitrary radii. [Symmetry codes: (i) x, -y + 3/2, z - 1/2; (ii) -x, -y + 2, -z; (iii) -x + 1, -y + 2, -z + 1; (iv) -x + 1, -y + 2, -z; (v) x - 1, y, z - 1.]

**Fig. 2.**
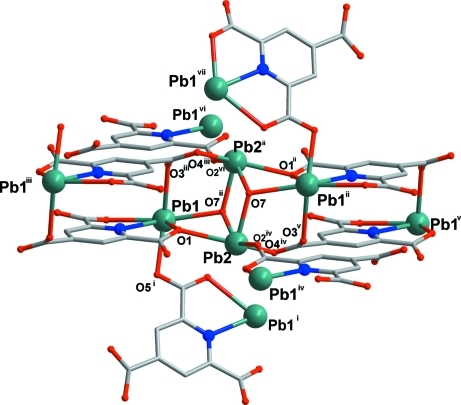
A view of the [Pb12Pb22(OH)2]6+ unit, surrounded by four Pb1 atoms. [Symmetry codes: (i) x, -y + 3/2, z - 1/2; (ii) -x, -y + 2, -z; (iii) -x + 1, -y + 2, -z + 1; (iv) -x + 1, -y + 2, -z; (v) x - 1, y, z - 1.]

**Fig. 3.**
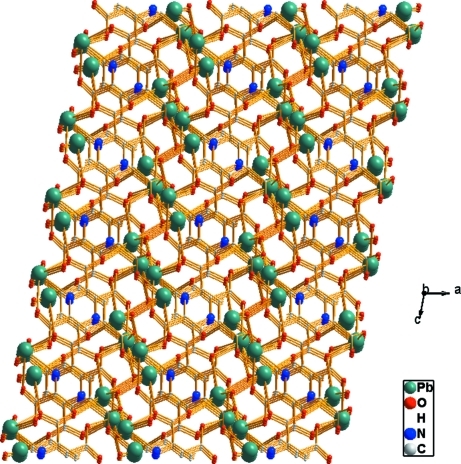
A packing diagram of the title compound viewed along the b axis.

## supplementary crystallographic information

### Comment

Until recently, the construction of coordination polymers has been an active area because of the properties of catalysis and molecular magnetism (Shi *et al.*, 2010; O'Keeffe *et al.*, 2008; Zhang *et al.*, 2009). Pyridine-2,4,6-tricarboxylic acid (H_3_ptc) is an effective ligand for coordinating to metal cations to generate diverse interesting coordination polymer architectures (Zhao *et al.*, 2009; Yigit *et al.*, 2005). However, the coordination polymers containing H_3_ptc ligands are seldom high-dimensional complexes (Das *et al.*, 2009; Ghosh *et al.*, 2006). Because of the relatively large ionic radius of the Pb(II) cation, the lead complex should form some interesting frameworks (Ding *et al.*, 2009). Herein, we report the lead polymeric complex [Pb_2_(C_8_H_2_NO_6_)(OH)]*_n_*, (I), which is an unique homometallic three-dimensional framework compound.

The asymmetric unit of (I) consists of two Pb(II) cations, one ptc trianion and one coordinated hydroxyl anion. As shown in Fig. 1, atom Pb1 is six-coordinated by two carboxylate O and one N atoms from a ligand ptc and one hydroxide anion O in a distorted square-planar geometry, and two carboxylate O atoms from the other two ligand ptc in the axial positions (Table 1). The PbNO_5_ octahedron is distorted, with the O—Pb1—O(N) bond angles ranging from 64.0 (2) to 147.70 (19)°. Whereas atom Pb2 is five-coordinated by three carboxylate O atoms from two ptc and two µ_3_-hydroxide O atoms in a distorted tetragonal pyramid geometry. The Pb—O and Pb—N distances (Table 1) are comparable with those observed for [Pb_2_(bdc)_2_(piphen)_2_]*_n_* (bdc is benzene-1,4-dicarboxylate and piphen is 6-(4-pyridyl)-5*H*- imidazolo[4,5-*f*][1,10]phenanthroline; Xu *et al.*, 2010). Each ptc molecule employs its three carboxylate groups and one N atom to chelate and bridge six Pb(II) cations. Two neighbouring Pb2 atoms are bis-bridged by two hydroxide O atoms (O7 and O7^ii^) to form a centrosymmetric [Pb_2_(OH)_2_]^2+^ core which is linked by two Pb1 atoms through Pb1—O7^ii^ and Pb1^ii^—O7 bonds to form [Pb1_2_Pb2_2_(OH)_2_]^6+^ unit (Fig. 2). The bonds of Pb2—O4^iv^, Pb2—O1, Pb2^ii^—O1^ii^ and Pb2^ii^—O4^iii^ bonds surrounding the [Pb_2_(OH)_2_]^2+^ unit are contributed to forming a two-dimensional structure which is further tightened by the atoms of Pb1^i^, Pb1^iii^, Pb1^iv^, Pb1^v^, Pb1^vi^ and Pb1^vii^ with joining neighbouring ptc ligands. Also, the two adjacent pyridine rings of ptc ligands are involved in π–π stacking interactions with offset face-to-face mode [centroid-to-centroid distance 3.5486 (4) Å] (Fig. 2). Furthermore, the two-dimensional structure are linked through Pb1—O5^i^ and Pb1^ii^—O5^v^ bonds to generate a three-dimensional stereo structure. There are some hydrogen bonds O—H···O in (I) between the hydroxide H atom and carboxylic O4^iii^ with an O···O distance of 2.884 (8) Å (Table 2). Hydrogen bonds are helpful to enhance the stability of the molecular structure. A remarkable feature of this structure is the arrangement of [Pb1_2_Pb2_2_(OH)_2_]^6+^ units with infinite helices extending along the crystallographic *b* axis with intervening ptc ligands (Fig. 3). The helical structure is a comprehensive result of metal-ligand interactions and the π–π stacking interactions of pyridine rings of ptc ligands.

### Experimental

A solution of pyridine-2,4,6-tricarboxylic acid (208 mg, 1.0 mmol) and KOH (224 mg, 4.0 mmol) in anhydrous methanol (10 ml) was added slowly to a solution of Pb(CH_3_COO)_2_ (672 mg, 2.0 mmol) in anhydrous methanol (10 ml). The resulting mixture was stirred for about 1 h at room temperature, sealed in a 25 ml Teflon-lined stainless steel autoclave and heated at 393 K for five days under autogenous pressure. The reaction system was cooled gradually to room temperature and colorless block-shaped crystals suitable for X-ray diffraction were collected.

### Refinement

H atoms were positioned geometrically (C—H = 0.93 Å and O—H = 0.83 Å) and included in the refinement in the riding-model approximation, with *U*_iso_(H) =1.2*U*_eq_(C) and 1.5*U*_eq_(O). The highest peak and the deepest hole in the difference Fourier map are located 0.78 and 0.97 Å, respectively, from atom Pb2.

### Crystal data

**Table:**

[Pb_2_(C_8_H_2_NO_6_)(OH)]	*F*(000) = 1112
*M**_r_* = 639.49	*D*_x_ = 3.918 Mg m^−^^3^
Monoclinic, *P*2_1_/*c*	Mo *K*α radiation, λ = 0.71073 Å
Hall symbol: -P 2ybc	Cell parameters from 5542 reflections
*a* = 7.5391 (9) Å	θ = 2.5–28.2°
*b* = 14.1845 (17) Å	µ = 31.05 mm^−^^1^
*c* = 10.3084 (12) Å	*T* = 291 K
β = 100.468 (1)°	Block, white
*V* = 1084.0 (2) Å^3^	0.38 × 0.26 × 0.25 mm
*Z* = 4	

### Data collection

**Table:**

Bruker SMART APEX CCD area-detector diffractometer	2014 independent reflections
Radiation source: fine-focus sealed tube	1903 reflections with *I* > 2σ(*I*)
graphite	*R*_int_ = 0.036
φ and ω scans	θ_max_ = 25.5°, θ_min_ = 2.5°
Absorption correction: multi-scan (*SADABS*; Sheldrick, 1996)	*h* = −9→9
*T*_min_ = 0.030, *T*_max_ = 0.047	*k* = −17→17
7870 measured reflections	*l* = −12→12

### Refinement

**Table:**

Refinement on *F*^2^	Primary atom site location: structure-invariant direct methods
Least-squares matrix: full	Secondary atom site location: difference Fourier map
*R*[*F*^2^ > 2σ(*F*^2^)] = 0.028	Hydrogen site location: inferred from neighbouring sites
*wR*(*F*^2^) = 0.069	H-atom parameters constrained
*S* = 1.08	*w* = 1/[σ^2^(*F*_o_^2^) + (0.0382*P*)^2^ + 7.726*P*] where *P* = (*F*_o_^2^ + 2*F*_c_^2^)/3
2014 reflections	(Δ/σ)_max_ = 0.001
157 parameters	Δρ_max_ = 1.64 e Å^−^^3^
0 restraints	Δρ_min_ = −2.07 e Å^−^^3^

### Special details

**Table:**

Geometry. All e.s.d.'s (except the e.s.d. in the dihedral angle between two l.s. planes)are estimated using the full covariance matrix. The cell e.s.d.'s are takeninto account individually in the estimation of e.s.d.'s in distances, anglesand torsion angles; correlations between e.s.d.'s in cell parameters are onlyused when they are defined by crystal symmetry. An approximate (isotropic)treatment of cell e.s.d.'s is used for estimating e.s.d.'s involving l.s. planes.
Refinement. Refinement of *F*^2^ against ALL reflections. The weighted *R*-factor *wR* and goodness of fit *S* are based on *F*^2^, conventional *R*-factors *R* are based on *F*, with *F* set to zero for negative *F*^2^. The threshold expression of *F*^2^ > σ(*F*^2^) is used only for calculating *R*-factors(gt) *etc*. and is not relevant to the choice of reflections for refinement. *R*-factors based on *F*^2^ are statistically about twice as large as those based on *F*, and *R*- factors based on ALL data will be even larger.

### Fractional atomic coordinates and isotropic or equivalent isotropic displacement parameters (Å^2^)

**Table:**

	*x*	*y*	*z*	*U*_iso_*/*U*_eq_	
Pb1	0.06507 (4)	0.84421 (2)	0.28447 (3)	0.01366 (11)	
Pb2	0.18306 (4)	0.93099 (2)	−0.06649 (3)	0.01411 (11)	
O1	0.2977 (9)	0.9297 (5)	0.1872 (6)	0.0289 (11)	
O2	0.5925 (8)	0.9461 (5)	0.1816 (6)	0.0289 (11)	
O3	0.9877 (8)	0.9721 (4)	0.6667 (6)	0.0225 (13)	
O4	0.9167 (8)	0.8519 (4)	0.7875 (6)	0.0250 (14)	
O5	0.2690 (8)	0.7768 (4)	0.7342 (6)	0.0213 (12)	
O6	0.1106 (8)	0.7676 (4)	0.5298 (5)	0.0229 (13)	
O7	0.0597 (7)	1.0796 (4)	−0.0536 (5)	0.0152 (11)	
H7	0.1117	1.1285	−0.0232	0.023*	
N1	0.3717 (8)	0.8650 (4)	0.4344 (6)	0.0105 (12)	
C1	0.5100 (10)	0.9033 (5)	0.3862 (7)	0.0112 (14)	
C2	0.6798 (11)	0.9170 (5)	0.4616 (8)	0.0162 (16)	
H2	0.7720	0.9438	0.4249	0.019*	
C3	0.7094 (10)	0.8896 (5)	0.5943 (8)	0.0140 (15)	
C4	0.5682 (11)	0.8484 (5)	0.6457 (8)	0.0175 (17)	
H4	0.5847	0.8288	0.7331	0.021*	
C5	0.4032 (10)	0.8379 (5)	0.5626 (8)	0.0116 (15)	
C6	0.4651 (11)	0.9281 (5)	0.2410 (8)	0.0150 (16)	
C7	0.8872 (11)	0.9068 (6)	0.6901 (8)	0.0163 (16)	
C8	0.2459 (11)	0.7909 (5)	0.6105 (7)	0.0147 (16)	

### Atomic displacement parameters (Å^2^)

**Table:**

	*U*^11^	*U*^22^	*U*^33^	*U*^12^	*U*^13^	*U*^23^
Pb1	0.01119 (17)	0.01504 (17)	0.01652 (18)	−0.00022 (11)	0.00723 (12)	0.00207 (10)
Pb2	0.01304 (18)	0.01647 (18)	0.01407 (17)	0.00281 (11)	0.00580 (12)	0.00070 (10)
O1	0.018 (2)	0.048 (3)	0.021 (2)	−0.010 (2)	0.0067 (19)	0.010 (2)
O2	0.018 (2)	0.048 (3)	0.021 (2)	−0.010 (2)	0.0067 (19)	0.010 (2)
O3	0.013 (3)	0.022 (3)	0.032 (3)	−0.007 (3)	0.004 (3)	−0.010 (3)
O4	0.019 (3)	0.037 (4)	0.019 (3)	0.003 (3)	0.002 (2)	0.002 (3)
O5	0.023 (3)	0.025 (3)	0.018 (3)	−0.003 (3)	0.010 (2)	0.003 (2)
O6	0.021 (3)	0.030 (3)	0.019 (3)	−0.017 (3)	0.006 (3)	−0.002 (2)
O7	0.014 (3)	0.013 (3)	0.021 (3)	−0.002 (2)	0.008 (2)	−0.001 (2)
N1	0.010 (3)	0.008 (3)	0.016 (3)	−0.002 (2)	0.006 (3)	0.001 (2)
C1	0.012 (4)	0.009 (3)	0.014 (4)	−0.001 (3)	0.008 (3)	0.001 (3)
C2	0.016 (4)	0.012 (4)	0.023 (4)	0.001 (3)	0.008 (3)	−0.002 (3)
C3	0.011 (4)	0.008 (3)	0.024 (4)	−0.003 (3)	0.005 (3)	−0.004 (3)
C4	0.017 (4)	0.019 (4)	0.016 (4)	−0.002 (3)	0.002 (3)	0.001 (3)
C5	0.011 (4)	0.009 (4)	0.016 (4)	0.000 (3)	0.003 (3)	−0.003 (3)
C6	0.012 (4)	0.019 (4)	0.015 (4)	−0.005 (3)	0.005 (3)	0.003 (3)
C7	0.017 (4)	0.017 (4)	0.016 (4)	0.009 (3)	0.005 (3)	−0.002 (3)
C8	0.023 (4)	0.008 (3)	0.015 (4)	0.000 (3)	0.009 (3)	−0.001 (3)

### Geometric parameters (Å, °)

**Table:**

Pb1—O5^i^	2.422 (6)	O5—C8	1.272 (9)
Pb1—O1	2.489 (6)	O5—Pb1^vii^	2.422 (6)
Pb1—N1	2.554 (6)	O6—C8	1.238 (10)
Pb1—O7^ii^	2.627 (5)	O7—Pb2^ii^	2.393 (5)
Pb1—O3^iii^	2.697 (6)	O7—Pb1^ii^	2.627 (5)
Pb1—O6	2.716 (6)	O7—H7	0.8286
Pb2—O1	2.600 (6)	N1—C1	1.348 (10)
Pb2—O2^iv^	2.836 (7)	N1—C5	1.355 (10)
Pb2—O4^v^	2.541 (6)	C1—C2	1.385 (11)
Pb2—O7	2.318 (5)	C1—C6	1.515 (10)
Pb2—O7^ii^	2.393 (5)	C2—C3	1.401 (11)
O1—C6	1.283 (10)	C2—H2	0.9300
O2—C6	1.256 (10)	C3—C4	1.400 (12)
O3—C7	1.247 (11)	C3—C7	1.533 (11)
O3—Pb1^iii^	2.698 (6)	C4—C5	1.384 (11)
O4—C7	1.258 (10)	C4—H4	0.9300
O4—Pb2^vi^	2.541 (6)	C5—C8	1.519 (11)
			
O5^i^—Pb1—O1	74.8 (2)	Pb2—O7—H7	127.9
O5^i^—Pb1—N1	70.8 (2)	Pb2^ii^—O7—H7	102.2
O1—Pb1—N1	64.0 (2)	Pb1^ii^—O7—H7	93.8
O5^i^—Pb1—O7^ii^	103.54 (18)	C1—N1—C5	117.6 (6)
O1—Pb1—O7^ii^	66.24 (19)	C1—N1—Pb1	119.9 (5)
N1—Pb1—O7^ii^	129.54 (18)	C5—N1—Pb1	122.5 (5)
O5^i^—Pb1—O3^iii^	147.70 (19)	N1—C1—C2	123.2 (7)
O1—Pb1—O3^iii^	75.1 (2)	N1—C1—C6	114.1 (6)
N1—Pb1—O3^iii^	85.62 (18)	C2—C1—C6	122.6 (7)
O7^ii^—Pb1—O3^iii^	74.41 (17)	C1—C2—C3	118.4 (7)
O5^i^—Pb1—O6	86.45 (19)	C1—C2—H2	120.8
O1—Pb1—O6	126.23 (19)	C3—C2—H2	120.8
N1—Pb1—O6	62.30 (18)	C4—C3—C2	119.2 (7)
O7^ii^—Pb1—O6	166.35 (17)	C4—C3—C7	117.3 (7)
O3^iii^—Pb1—O6	102.15 (18)	C2—C3—C7	123.4 (7)
O7—Pb2—O7^ii^	71.0 (2)	C5—C4—C3	118.1 (7)
O7—Pb2—O4^v^	98.8 (2)	C5—C4—H4	121.0
O7^ii^—Pb2—O4^v^	71.47 (19)	C3—C4—H4	121.0
O7—Pb2—O1	90.7 (2)	N1—C5—C4	123.5 (7)
O7^ii^—Pb2—O1	67.99 (19)	N1—C5—C8	115.6 (7)
O4^v^—Pb2—O1	132.45 (19)	C4—C5—C8	120.9 (7)
C6—O1—Pb1	121.8 (5)	O2—C6—O1	124.4 (7)
C6—O1—Pb2	123.8 (5)	O2—C6—C1	118.4 (7)
Pb1—O1—Pb2	106.2 (2)	O1—C6—C1	117.2 (7)
C7—O3—Pb1^iii^	124.4 (5)	O3—C7—O4	126.0 (8)
C7—O4—Pb2^vi^	101.9 (5)	O3—C7—C3	119.0 (7)
C8—O5—Pb1^vii^	110.5 (5)	O4—C7—C3	115.0 (7)
C8—O6—Pb1	117.9 (5)	O6—C8—O5	125.3 (7)
Pb2—O7—Pb2^ii^	108.9 (2)	O6—C8—C5	119.7 (7)
Pb2—O7—Pb1^ii^	113.8 (2)	O5—C8—C5	115.0 (7)
Pb2^ii^—O7—Pb1^ii^	108.2 (2)		
			
O5^i^—Pb1—O1—C6	−61.3 (6)	C5—N1—C1—C6	177.8 (6)
N1—Pb1—O1—C6	14.5 (6)	Pb1—N1—C1—C6	−1.6 (8)
O7^ii^—Pb1—O1—C6	−173.9 (7)	N1—C1—C2—C3	0.1 (11)
O3^iii^—Pb1—O1—C6	106.9 (6)	C6—C1—C2—C3	−178.4 (7)
O6—Pb1—O1—C6	12.5 (7)	C1—C2—C3—C4	0.8 (11)
O5^i^—Pb1—O1—Pb2	88.2 (3)	C1—C2—C3—C7	−175.9 (7)
N1—Pb1—O1—Pb2	163.9 (3)	C2—C3—C4—C5	−0.9 (11)
O7^ii^—Pb1—O1—Pb2	−24.43 (19)	C7—C3—C4—C5	175.9 (7)
O3^iii^—Pb1—O1—Pb2	−103.7 (3)	C1—N1—C5—C4	0.6 (11)
O6—Pb1—O1—Pb2	162.02 (19)	Pb1—N1—C5—C4	180.0 (6)
O7—Pb2—O1—C6	−115.5 (6)	C1—N1—C5—C8	−177.3 (6)
O7^ii^—Pb2—O1—C6	175.3 (7)	Pb1—N1—C5—C8	2.1 (8)
O4^v^—Pb2—O1—C6	141.8 (6)	C3—C4—C5—N1	0.2 (11)
O7—Pb2—O1—Pb1	95.8 (3)	C3—C4—C5—C8	178.0 (7)
O7^ii^—Pb2—O1—Pb1	26.6 (2)	Pb1—O1—C6—O2	159.2 (7)
O4^v^—Pb2—O1—Pb1	−6.8 (4)	Pb2—O1—C6—O2	15.2 (11)
O5^i^—Pb1—O6—C8	82.5 (6)	Pb1—O1—C6—C1	−21.2 (9)
O1—Pb1—O6—C8	14.3 (7)	Pb2—O1—C6—C1	−165.2 (5)
N1—Pb1—O6—C8	12.3 (5)	N1—C1—C6—O2	−165.9 (7)
O7^ii^—Pb1—O6—C8	−139.9 (7)	C2—C1—C6—O2	12.7 (12)
O3^iii^—Pb1—O6—C8	−66.1 (6)	N1—C1—C6—O1	14.5 (10)
O7^ii^—Pb2—O7—Pb2^ii^	0.001 (1)	C2—C1—C6—O1	−167.0 (7)
O4^v^—Pb2—O7—Pb2^ii^	66.8 (2)	Pb1^iii^—O3—C7—O4	−100.9 (9)
O1—Pb2—O7—Pb2^ii^	−66.4 (2)	Pb1^iii^—O3—C7—C3	78.7 (8)
O7^ii^—Pb2—O7—Pb1^ii^	−120.9 (3)	Pb2^vi^—O4—C7—O3	11.2 (9)
O4^v^—Pb2—O7—Pb1^ii^	−54.0 (2)	Pb2^vi^—O4—C7—C3	−168.5 (5)
O1—Pb2—O7—Pb1^ii^	172.7 (2)	C4—C3—C7—O3	−152.7 (7)
O5^i^—Pb1—N1—C1	76.4 (5)	C2—C3—C7—O3	24.0 (11)
O1—Pb1—N1—C1	−5.6 (5)	C4—C3—C7—O4	27.0 (10)
O7^ii^—Pb1—N1—C1	−15.5 (6)	C2—C3—C7—O4	−156.3 (7)
O3^iii^—Pb1—N1—C1	−81.2 (5)	Pb1—O6—C8—O5	165.4 (6)
O6—Pb1—N1—C1	172.7 (6)	Pb1—O6—C8—C5	−16.6 (9)
O5^i^—Pb1—N1—C5	−103.0 (5)	Pb1^vii^—O5—C8—O6	18.5 (10)
O1—Pb1—N1—C5	175.0 (6)	Pb1^vii^—O5—C8—C5	−159.6 (5)
O7^ii^—Pb1—N1—C5	165.1 (5)	N1—C5—C8—O6	10.3 (10)
O3^iii^—Pb1—N1—C5	99.5 (5)	C4—C5—C8—O6	−167.6 (7)
O6—Pb1—N1—C5	−6.7 (5)	N1—C5—C8—O5	−171.5 (6)
C5—N1—C1—C2	−0.7 (11)	C4—C5—C8—O5	10.6 (11)
Pb1—N1—C1—C2	179.8 (6)		

Symmetry codes: (i) *x*, −*y*+3/2, *z*−1/2; (ii) −*x*, −*y*+2, −*z*; (iii) −*x*+1, −*y*+2, −*z*+1; (iv) −*x*+1, −*y*+2, −*z*; (v) *x*−1, *y*, *z*−1; (vi) *x*+1, *y*, *z*+1; (vii) *x*, −*y*+3/2, *z*+1/2.

### Hydrogen-bond geometry (Å, °)

**Table:**

*D*—H···*A*	*D*—H	H···*A*	*D*···*A*	*D*—H···*A*
O7—H7···O6^viii^	0.83	2.58	2.989 (8)	112.
O7—H7···O4^iii^	0.83	2.49	2.884 (8)	110.

Symmetry codes: (viii) −*x*, *y*+1/2, −*z*+1/2; (iii) −*x*+1, −*y*+2, −*z*+1.
